# Children Coping, Contextual Risk and Their Interplay During the COVID-19 Pandemic: A Spanish Case

**DOI:** 10.3389/fpsyg.2020.577763

**Published:** 2020-12-16

**Authors:** Beatriz Domínguez-Álvarez, Laura López-Romero, Aimé Isdahl-Troye, Jose Antonio Gómez-Fraguela, Estrella Romero

**Affiliations:** Department of Clinical Psychology and Psychobiology, Universidade de Santiago de Compostela, Santiago de Compostela, Spain

**Keywords:** children coping, pandemic, COVID-19-related stressors, adjustment, parent resilience

## Abstract

The COVID-19 pandemic has changed the lives of millions of people around the globe and some of the unprecedent emerged disruptions, are likely to have been particularly challenging for young children (e.g., school closures, social distancing measures, movement restrictions). Studying the impact of such extraordinary circumstances on their well-being is crucial to identify processes leading to risk and resilience. To better understand how Spanish children have adapted to the stressful disruptions resulting from the pandemic outbreak, we examined the effects of child coping and its interactions with contextual stressors (pandemic and family related) on child adjustment, incorporating in our analysis a developmental perspective. Data was collected in April 2020, through parent-reports, during the acute phase of the pandemic and, temporarily coinciding with the mandatory national quarantine period imposed by the Spanish Government. A sample of 1,123 Spanish children (50% girls) aged 3 to 12 (Mage = 7.26; SD = 2.39) participated in the study. Results showed differences in the use of specific strategies by children in different age groups (i.e., 3–6, 7–9 and 10–12-year-olds). Despite the uncontrollable nature of the pandemic-related stressors, child disengagement coping was distinctively associated to negative outcomes (i.e., higher levels of behavioral and emotional difficulties), whereas engagement coping predicted psychosocial adjustment across all age groups. Moreover, interactively with child coping, parent fear of the future and parent dispositional resilience appear as relevant contextual factors to predict both negative and positive outcomes, but their effects seem to be age dependent, suggesting a higher contextual vulnerability for younger children. These findings might have implications for identifying individual and contextual risk and informing potential preventive interventions aimed to reduce the impact of future pandemic outbreaks on children of different ages.

## Introduction

The global crisis originated by the recent COVID-19 pandemic is not comparable, neither in magnitude nor in kind, to any other similar experienced before (e.g., SARS- outbreak, Prime et al., 2020). It is unprecedent as, for the first time, we are exposed to a considerable number of unfamiliar stressors (e.g., social distancing, restriction of movement; Yan, 2020), acutely emerged, but timely sustained by public preventive health measures imposed world-wide (i.e., mandatory home confinement). The psychological long-term effects of these measures remain, currently, largely unknown (Brooks et al., 2020; Green, 2020).

Children, too, have been exposed to these and other aged-related specific stressors (e.g., school closures and online homeschooling, The Lancet Child Adolescent Health, 2020). Only recently, some empirical evidence relative to the negative effects on children adjustment is beginning to be gathered from studies conducted, mainly, in affected developed countries. Altogether, these preliminary findings point out to an increased risk of experiencing negative consequences such as depressive and anxiety symptoms (Xie et al., 2020) and changes in emotional states and behaviors (e.g., difficulty concentrating, boredom, irritability, Orgilés et al., 2020). However, at this point, the literature on the impact of COVID-19 pandemic on children psychosocial well-being is still very scarce and the insights from previous pandemic experiences quite limited (Koller et al., 2010; Murray, 2010; Sprang and Silman, 2015).

At the same time, there is enough evidence from developmental, preventive and clinical literature suggesting that children’s adjustment to these extraordinary social disruptions is likely to be multi-determined by individual and environmental factors, whose effects might contribute to short and long-term adaptation (Compas, 2006; Grant et al., 2006; Luthar, 2006; Cicchetti, 2010; Blair and Raver, 2012).

First, the ability to effectively cope with the stressful situation, that is to “mobilize, modulate, manage, and coordinate the own behavior, emotions and attention under stress” (p. 6, Skinner and Zimmer-Gembeck, 2009), has been consistently associated to child adjustment in diverse difficult circumstances and at different ages (Smith et al., 2006; Cicchetti and Rogosch, 2009; Zimmer-Gembeck and Skinner, 2011a; Terranova et al., 2015). In children coping research, at a broader level, engagement and disengagement coping (i.e., oriented toward or away the source of stress and/or one’s emotions and thoughts) are distinctively associated to different outcomes (Compas et al., 2001). However, these associations seem to be dependent on the controllable or uncontrollable nature of the stressful events (Altshuler and Ruble, 1989; Compas et al., 1991; Clarke, 2006; Wadsworth, 2015). For instance, under controllable stressful conditions, engagement coping is predictive of lower levels of internalizing and externalizing symptoms, whereas disengagement coping, on the contrary, contributes to higher levels of these difficulties (Connor-Smith et al., 2000). However, an avoidant-distractive coping was linked to less negative emotions and short-term maladjustment when children must deal, respectively, with uncontrollable medical stressors (Band and Weisz, 1988) and family marital conflict (O’Brien et al., 1995). Interestingly, there is also evidence suggesting that emotional disengagement (i.e., attempting to eliminate subjective feelings and outward signs of emotion) is a useful short-term strategy for regulating negative emotion (Rice et al., 2007). Applying an engaged-oriented coping to uncontrollable conditions, could increase psychological distress due to the inefficacy of these strategies to modify the objective stressful conditions or maximize one goodness of fit with them as they are (Yeo et al., 2014). Hence, these findings are, because of the very nature of the current widespread pandemic, of much relevance.

In addition, distal and proximal contextual influences can independently affect child adjustment but also moderate the coping-outcomes relationship (Compas, 2009; Main et al., 2011). Globally, despite individual differences at micro-level contexts, children and their families were exposed, world-wide, to mild-moderate levels of stress resulting from potentially experiencing multiple and unique COVID-19-related stressors. Beyond the domain of individual physical health and psychological well-being (e.g., COVID-19 contagion, Guo et al., 2020), the economic capacity and financial security (e.g., job loss, Baker et al., 2020) and family dynamics (e.g., parent-child relation, Cluver et al., 2020; Prime et al., 2020) were also unexpectedly and profoundly altered. Thus, the way in which parents respond to these disruptive experiences is also essential to define the impact of COVID-19 crisis on children, as for it directly influences not only the youngsters’ response to normative stressors (Kliewer et al., 1996) but also to extremely challenging conditions (e.g., natural disasters, war; Bradley, 2007). Modeling processes are one of the core mechanisms through which parents contribute to children’s response to stress (Power, 2004). In fact, a positive model of coping, along with the exposure to mild-moderate stress levels and an age-appropriate scaffolding are necessary conditions for the children to develop a healthy repertoire of coping skills (Wadsworth, 2015). Thus, it is likely to be the case that a relatively stable and consistent parental disposition to resist adverse circumstances (i.e., trait resilience, Almedom and Glandon, 2007; Connor and Davidson, 2003) would affect the children capacity to cope under adverse conditions. Moreover, besides these vicarious influences, the use of ineffective coping strategies could also directly undermine parent individual well-being and consequently affect child well-being, by damaging the parent-child relation during the COVID-19 pandemic (Prime et al., 2020). For these reasons, the current scenario offers a unique opportunity to study these interactive coping-context relations and the moderator effect of distal and immediate contextual factors on coping-outcomes relation.

Finally, along with these environmental transactions, children’s ability to successfully cope with adverse and stressful conditions is primarily shaped and constrained by their cognitive and emotional development (Skinner and Zimmer-Gembeck, 2009). Child developmentally determined skills and capacities account not only for the differences in the type of coping strategies displayed by children in different age groups but also for the age-dependent normative shifts occurring at certain points (Skinner and Zimmer-Gembeck, 2016a). In fact, their progressive acquisition explains the quantitative and qualitative normative changes in the development of coping occurring during critical transitions (e.g., in infancy to toddlerhood, between ages 5–7 or in late childhood to early adolescence, Skinner and Zimmer-Gembeck, 2007). However, despite the wide recognition of this need to adopt a developmental perspective in the study of children coping, and the evidence supporting the feasibility of its empirical examination even at very early ages (i.e., preschool years, Yeo et al., 2014), these developmental considerations have been rarely incorporated into the empirical research (Compas et al., 2001; Skinner and Zimmer-Gembeck, 2007, 2009).

The main goal of this study is to examine how child coping, unique contextual conditions and parent dispositional resilience, might contribute to the children psychosocial adjustment during the extraordinary context of COVID-19 pandemic, incorporating in our analysis a developmental perspective. Specifically, we aimed (1) to explore the use of specific strategies and broad dimensions of coping by children of different age-groups (2) to determine the positive and negative outcomes of child engagement and disengagement coping under uncontrollable circumstances, (3) to examine to which extent the relation between coping and outcomes is moderated by situational stressors specifically related to the current crisis, (4) to analyze if contextual factors within the family system (i.e., parental resilience) also moderate the coping-outcomes relation and finally (5) to test if these effects are age-dependent. We hypothesized, based on the literature reviewed, that the main coping strategies and dimensions used by children would differ between age groups. Also, the associations of engagement and disengagement coping with child adjustment might be different to the often observed under controllable stressful situations, but distal and proximal contextual factors would moderate the coping-outcomes relation anyhow. Finally, these moderation effects could be age dependent.

## Materials and Methods

### Participants

Data for the current research is from the CONFIA-20 Study (*Confinement Effects on Families and Children*), which was aimed to examine the psychological, emotional and behavioral effects of home-confinement in children and families from the region of Galicia, NW Spain. It was conducted in April 2020, during the acute phase of COVID-19 pandemic, temporarily coinciding with the mandatory national quarantine period imposed to the whole population by the Spanish Government from March13th to April 26th of 2020. Children and families considered to participate in this study had to live in Spain. Despite Galicia being the primary geographical area of interest, families from other Spanish regions were allowed to participate. Families from other countries and/or continents were excluded. Also, parents had to fill the questionnaire strictly within the temporal limits of the national quarantine period, that is, never after April 27th of 2020. Finally, the “target-child” to whom the questionnaire was referred to, could not be younger than three or older than twelve years old. The sample of 1,123 children (50% girls) aged 3–12 (Mage = 7.26; SD = 2.39) was composed by 481 preschoolers (aged 3–6; Mage = 4.95; SD = 0.93; 50.6% girls), 393 middle-aged children (aged 7–9; Mage = 7.98; SD = 0.83; 51.3% girls) and 248 early adolescents (aged 10–12; Mage = 10.62; SD = 0.67; 48.8% girls), when divided by meaningful age subgroups. Data collected was parent-reported (89.5% mothers) and most of the participating families were from Galicia (94.2%) with the remaining 5.8% from other Spanish regions (e.g., Madrid, Ciudad Real, Barcelona, Cantabria, Zamora). Most of the parents were working before the crisis (86.9%) and, at the time of data collection, almost a half of them (46.9%) declared no difficulties making ends meet. Among those previously employed, they globally maintained their jobs (17.9% kept attending, 33.9% kept working from home, 19.1% were on temporary stoppages, 8.6% were on a medical leave), whereas 2.4% of them lost their employment due to the COVID-19 crisis. At the time of data collection, families had been 30.87 (SD = 6.37) days confined, at homes of around 126.68 square meters (ranging from 35 to 1,600; 50.7% of them with garden), with about four people per home (M = 3.90, SD = 1.01). Finally, 16.7% of the participating parents reported the existence of COVID-19 contagion cases in their close social circle (i.e., family and friends) and 5.3% of them informed of close COVID-19 related deaths (for a detailed characterization of the sample see Table 1).

**TABLE 1:** Descriptive statistics of the CONFIA-20 study sample: COVID-19-related stressors, demographics variables of participating parents, children and family relevant domains.

	Total (*N* = 1123)	Range
Person filling the questionnaire: Mother, *N* (%)	1004(89.5%)	
**COVID-19 crisis related stressors**		
Number of days of confinement, *M* (*SD*)	30.57 (6.47)	0–60
Number of people during confinement, *M* (*SD*)	3.89 (1.01)	1–10
House dimensions in square meters, *M* (*SD*)	126.68 (93.98)	35–1600
House with garden: Yes, *N* (%)	567 (50.7)	0–1
COVID-19 contagion in close circle (family, friends), *N* (*%*)	187 (16.7)	0–1
COVID-19 related death in close circle (family, friends), *N* (*%*)	59 (5.3)	0–1
Perceived economic impact on the family, *M* (*SD*)	1.36 (1.00)	0–3
Negative influence of confinement on family relations, *M* (*SD*)	0.80 (0.78)	0–3
**Families**		
Geographic area of residency: Galicia, *N* (*%*)	899 (94.2)	–
Number of children per family, *M* (*SD*)	1.78 (0.69)	1–5
Parent perceived level of monthly income, *N* (*%*)		
Serious problems making ends meet	20 (1.8)	0–1
Difficulties making ends meet	90 (8.1)	0–1
Tightly making ends meet	483 (43.2)	0–1
Loosely making ends meet	524 (46.9)	0–1
SES, *M* (*SD*)	0.09 (0.7)	−2.6 to 1.3
Mother educational level, *N* (*%*)		
Doctoral or Master’s Degree	85 (7.6)	0–1
Undergraduate	608 (54.3)	0–1
Secondary school	333 (29.8)	0–1
Primary school	90 (8)	0–1
Mother current employment situation (mother-reported), *N* (*%*)		
Regular attendance	180 (18)	0–1
Work from home	319 (31.9)	0–1
Paralyzed working activity	195 (19.5)	0–1
Lost job due to COVID-19 crisis	25 (2.5)	0–1
Unemployed before the COVID-19 crisis	142 (14.2)	0–1
Father educational level, *N* (*%*)		
Doctoral or Master’s Degree	54 (4.9)	0–1
Undergraduate	381 (34.7)	0–1
Secondary school	437 (39.8)	0–1
Primary school	214 (19.5)	0–1
Father current employment situation (father-reported), *N* (*%*)		
Regular attendance	18 (16.2)	0–1
Work from home	58 (52.3)	0–1
Paralyzed working activity	18 (16.2)	0–1
Lost job due to COVID-19 crisis	1 (0.9)	0–1
Unemployed before the COVID-19 crisis	6 (5.4)	0–1
Optimal household resources (computer, wi-fi) for the children to do their schoolwork at home, *M* (*SD*)	2.84 (1.19)	0–4
**Children**		
Female, *N* (%)	551 (50)	0–1
Age, *M* (*SD*)	7.26 (2.4)	3–12
Medical or psychological difficulty: Yes, *N* (%)	141 (12.6)	0–1
Specific psychological difficulties		
TDAH, *N* (%)	25 (2.2)	0–1
TEA/Asperger, *N* (%)	12 (1.1)	0–1
Global adjustment to online home schooling, *M* (*SD*)	2.22 (1.1)	0–4
**Parent difficulties derived from COVID-19 crisis**		
Perceived level of stress, *M* (*SD*)	1.57 (0.79)	0–3
Reported fear of the future, *M* (*SD*)	1.78 (0.85)	0–3
Difficulties to reconcile working and family life *M* (*SD*)	2.27 (1.28)	0–4
Difficulties helping children with their academic tasks, *M* (*SD*)	2.91 (1.08)	0–4
Anxiety, *M* (*SD*)*	2.64 (0.71)	0–4
Depression, *M* (*SD*)*	2.39 (0.73)	0–4

*Items from the PHQ (Kroenke et al., 2009) were rated on a 5 point comparative scale from 0 (“much less”) to 4 (much more”) aiming to represent the change self-perceived in the emotional state as a result of the COVID-19 crisis.*

### Procedure

This study was conducted within the context of a large ongoing research, focused on studying child behavioral, emotional and social early development, and was approved by the Bioethics Committee of the University of Santiago de Compostela. We first developed a parent-reported questionnaire to be filled on an online secure platform. Then, we initiated a dissemination strategy by providing information of the study objectives and access to the survey link through (1) the official research group web page, (2) social media (3) telematic contacts with schools and parents associations and (4) informal diffusion actions. Data collection began at April 8th and ended at April 27th of 2020. Participation was anonymous and voluntary. Before filling the survey, parents gave their consent by explicitly agreeing to participate in the study. They were asked to refer their answers strictly to the COVID-19 situation crisis. The duration of the survey was around 15 min and participating families did not receive any reward or compensation.

### Measures and Instruments

**Child Coping.** We assessed context-specific coping on a parent-reported 22-item scale specifically developed for the CONFIA-20 study. After reviewing the available literature (Blount et al., 2008; Pfefferbaum et al., 2012), we selected, translated and adapted items from well-known children coping measures to be appropriate and relevant in content for the COVID-19 pandemic situation. We included items from the KidCOPE (13 items, Spirito et al., 1988) and The Children’s Coping Strategies Checklist (6 items, Ayers et al., 1996), plus 3 more *ad hoc* created items. Then, we grouped them into two broad categories (engagement and disengagement coping) on a conceptual basis, following the model of Connor-Smith et al. (2000). The resulting final scale was composed by two 11-item subscales assessing strategies such as “tries to calm him/herself” or “spontaneously proposes possible solutions to current crisis” (engagement coping) and “avoids thinking of the current situation” or “remains without doing anything because thinks that the current crisis cannot be solved” (disengagement coping). Parents rated the items on a 4-point Likert scale ranging from 0 (“never”) to 3 (“always or almost always”). The internal consistency of the engagement and disengagement scales was acceptable (α = 0.77 and α = 0.66, respectively). For further information about the specific items selected, see Supplementary Table 1.

**Child Maladjustment during the COVID-19 Pandemic.** We selected three subscales of the Strengths and Difficulties Questionnaire (SDQ, Goodman, 1997) to assess the negative consequences of COVID-19 crisis in child behavioral and emotional functioning. Specifically, conduct problems, hyperactive behaviors and emotional problems subscales. Examples of items selected are, respectively, “often has temper tantrums or hot tempers,” “is constantly fidgeting or squirming” or “is often unhappy, down-hearted or tearful.” The original 4-point scale response was adapted to a 5-point comparative format (0: much less, 1: some less, 2: no change, 3 some more, 4: much more), aiming to reflect the possible observed changes on child behavior compared to the pre-COVID-19 pandemic functioning. The internal consistency of the scales was acceptable (α = 0.81, α = 0. 61 and α = 0.77, respectively).

**Child Adjustment during the COVID-19 Pandemic.** We assessed the potential positive outcomes resulting from the pandemic crisis on a parent-reported 14-item scale specifically developed *ad hoc* for the CONFIA-20 study (see Romero et al., 2020 for further information about the scale). The four scales are routine maintenance (4 items; e.g., “he/she has adapted him/herself to a scheduled daily activity routine; α = 0.55), prosocial involvement (5 items; e.g., “shows interest to spare time with family”; α = 0.70), social-oriented reflection (3 items; e.g., “he/she assumes that we all should collaborate to slow down the pandemic”; α = 0.84), and social bonding (2 items; e.g., “keeps contact with his/her beloved ones who are not close, by phone, internet…”; α = 0.48). Parents rated each item on the same 5-point comparative scale used to assess negative outcomes.

**COVID-19 related Stressors.** *Ad hoc* items were created to asses CONFIA-20 participant parents’ experiences with COVID-19 related stressors. For the purposes of the current research, we have exclusively focused on health, financial and future-threatening acute stressors. They were assessed through single parent-reported items such as “I think that the COVID-19 crisis has damaged the economic situation of my family” or “the current crisis makes me fear the future” rated on a 4-point scale from 0 (“not at all”) to 3 (“very much”). Also, Yes/No independent questions asking for the existence of any COVID-19 related contagion and/or death on the social close circle were included. In case of an affirmative answer, parents were asked to detail the number of close contagions and/or deaths.

**Parent Resilience.** We used the 10-item version of the Connor-Davidson Resilience Scale (CD-RISC-10, Connor and Davidson, 2003; Campbell-Sills and Stein, 2007) to measure parental dispositional resilience. Items such as “I am able to adapt when changes occur,” “having to cope with stress can make me stronger” or “I try to see their humorous side when I am faced with problems” were rated by parents on a 4-point Likert scale ranging from 0 (“not at all”) to 4 (“true nearly all the time”). The CD-RISC-10 has been used in various samples (Wang et al., 2010; Notario-Pacheco et al., 2011) and in different cultures (Lauridsen et al., 2017) showing high reliability across studies. The internal consistency of the scale in our sample was excellent (α = 0.90).

**Covariate**s. We included an assessment of family socioeconomic status (SES), which was derived from questions about (1) parent education, (2) family income, and (3) family financial solvency to face daily overheads. Education level was computed as the mean of mother and father ratings on a six-point scale ranging from 1 (“without basic studies”) to 6 (“postgraduate”). Family income was based on parents’ reports of family income rated on a four-point scale from 1 (“serious problems making ends meet”) to 4 (“well off”). A composite SES was computed by first transforming the aforementioned variables into z-scores. Finally, we included child gender (0 = male, 1 = female) and age in years as covariates.

### Analytic Strategy

We first computed the descriptive statistics of the CONFIA-20 study sample, including the means, standard deviations and frequencies of COVID-19-related stressors, demographic variables of parents and children, and family relevant domains. We then ran an analysis of variance to compare the mean differences in broad dimensions of coping (engagement and disengagement) and fine-grained coping strategies by age groups (i.e., preschoolers, aged 3–6, children aged 7–9 and early adolescents, aged 10–12). Before the regression analyses, we explored the bivariate correlations between the study variables. All the above-mentioned analyses were conducted on SPSS Statistics version 26 (IBM Corp, 2019). Finally, on Mplus vs. 8.0 (Muthén and Muthén, 2019) we conducted multiple linear regression analysis to model the main effects of child coping on adjustment, controlling for other relevant variables such as sociodemographic covariates (i.e., sex, age, SES), COVID-19 related stressors (i.e., close contagion, close death, economic impact, fear of the future) and parental resilience. In the subsequent regression models, we included the interaction terms of the context-specific coping, with the contextual factors, to examine their potential moderating effects on the coping-outcomes relation. As it was hypothesized that these main and interactive associations could be age-dependent, complementary regression analysis, were conducted separately by age group subsamples.

## Results

### Age Differences in Coping

Overall, the context-specific coping of the CONFIA-20 children during the COVID-19 crisis was more engagement than disengagement-oriented (Table 2). In fact, a significant increase in the use of engagement strategies was found at the end of the preschool period. When analyzing the specific strategies used by the different age group subsamples (i.e., 3–6-year-olds, 7–9- year-olds and 10–12- year-olds) significant differences were found. Compared to older children, preschoolers tended to use more predominantly strategies such as “yelling or getting angry” (negative emotion regulation). Seven to nine year olds, however, seemed to start to display more engaged-oriented strategies such as “trying to do specific actions to solve the current crisis” (problem solving),“trying to understand how things like this happens”(seeking understanding) or “seeking help to try to improve the situation” (instrumental social support). Finally, early adolescents repertoire of behavioral and cognitive coping skills becomes not only more diverse [e.g., “making jokes or trying to laugh about the current situation” (humor) and “wishing it never had happened” (wishful thinking)] but also sustained in more complex regulatory capacities (e.g., “trying to calm him/herself,” positive emotion regulation).

**TABLE 2:** Total sample means and mean differences in broad dimensions of coping and specific coping strategies between children of different age groups.

CONFIA-20 Coping Scale items (Subscale)	Total sample *M* (*SD*)	Age group 1 (3–6 -year-olds) *M* (*SD*)	Age group 2 (7–9 -year-olds) *M* (*SD*)	Age group 3 (10–12- year-olds) *M* (*SD*)	Group differences *F* (*df*)	*p*	*Post hoc* comparisons
1. Seems to try to forget what is happening (DIS)	1.13 (0.96)	1.14 (0.99)	1.13 (1.00)	1.12 (0.85)	*F* (2,1116) 0.40	0.963	–
2. Tries to hold a positive view of the situation (ENG)	1.83 (0.91)	1.87 (0.93)	1.82 (0.88)	1.77 (0.91)	*F* (2,1117) = 1.04	0.353	–
3. Prefers to spend time alone (DIS)	0.62 (0.64)	0.51 (0.62)	0.61 (0.61)	0.84 (0.68)	*F* (2,1118) = 22.32	0.000	Sig: 1-2; 2-3
4. Blames someone for causing the current crisis (DIS)	0.25 (0.60)	0.24 (0.57)	0.24 (0.60)	0.30 (0.64)	*F* (2,1116) = 0.99	0.371	–
5. Spontaneously proposes possible solutions to current crisis (ENG)	0.80 (0.72)	0.79 (0.69)	0.87 (0.74)	0.73 (0.72)	*F* (2,1116) = 2.80	0.061	–
6. Yells or gets angry (DIS)	1.03 (0.73)	1.18 (0.73)	0.94 (0.69)	0.90 (0.70)	*F* (2,1118) = 17.87	0.000	Sig: 1-2; 1-3
7. Wishes the COVID-19 crisis had never happened (DIS)	1.71 (1.08)	1.59 (1.11)	1.80 (0.1.01)	1.83 (1.07)	*F* (2,1112) = 5.97	0.003	Sig: 1-2; 1-3
8. Spends time with other people (e.g., family members) (ENG)	1.95 (0.95)	1.97 (1.01)	1.99 (0.88)	1.84 (0.93)	*F* (2,1111) = 2.07	0.127	–
9. Does things (e.g., play or watch TV) to evade him/herself (DIS)	1.90 (0.95)	1.88 (0.99)	1.88 (0.92)	1.93 (0.89)	*F* (2,1112) = 0.27	0.759	–
10. Avoids talking about the COVID-19 pandemic (DIS)	0.77 (0.95)	0.75 (0.95)	0.74 (0.95)	0.86 (0.96)	*F* (2,1112) = 1.22	0.294	–
11. Tries to do specific actions to solve the current crisis (ENG)	0.99 (0.79)	0.89 (0.77)	1.07 (0.79)	1.03 (0.80)	*F* (2,1110) = 5.87	0.003	Sig: 1-2
12. Tries to calm him/herself (ENG)	1.00 (0.89)	0.91 (0.86)	1.04 (0.88)	1.13 (0.91)	*F* (2,1106) = 5.15	0.006	Sig: 1-3
13. Wishes something could be done to change the situation (DIS)	1.18 (0.94)	1.09 (0.95)	1.25 (0.91)	1.22 (0.94)	*F* (2,1108) = 3.48	0.031	Sig: 1-2
14. Remains without doing nothing (the situation can’t be solved) (DIS)	0.57 (0.87)	0.55 (0.88)	0.56 (0.84)	0.61 (0.88)	*F* (2,1102) = 0.42	0.657	–
15. Shares with us how she/he feels regarding the crisis (ENG)	1.16 (0.79)	1.09 (0.78)	1.21 (0.79)	1.22 (0.77)	*F* (2,1086) = 3.54	0.029	Sig: 1-3
16. Tries to understand how things like this happens (ENG)	1.36 (0.89)	1.28 (0.88)	1.42 (0.89)	1.44 (0.89)	*F* (2,1115) = 4.00	0.019	Sig: 1-2
17. Makes jokes or tries to laugh about the current situation (ENG)	0.75 (0.80)	0.66 (0.76)	0.79 (0.83)	0.85 (0.80)	*F* (2,1115) = 5.50	0.004	Sig: 1-3
18. Seeks help in others to understand what is happening (ENG)	1.27 (0.83)	1.28 (0.84)	1.29 (0.83)	1.22 (0.81)	*F* (2,1116) = 0.56	0.570	–
19. Reminds him/herself that his/her situation is not that bad (ENG)	1.41 0.98)	1.27 (1.02)	1.51 (0.95)	1.53 (0.91)	*F* (2,1103) = 8.87	0.000	Sig: 1-2; 1-3
20. Avoids thinking about the current crisis (DIS)	1.11 (0.89)	1.06 (93.)	1.14 (0.89)	1.13 (0.83)	*F* (2,1103) = 0.90	0.408	–
21. Seeks help to try to improve the situation (ENG)	0.96 (0.79)	0.09 (0.81)	1.07 (0.81)	0.92 (0.72)	*F* (2,1105) = 5.30	0.005	Sig: 1-2
22. Fantasizes with a prompt resolution for the current crisis (DIS)	1.50 (0.92)	1.55 (0.91)	1.47 (0.95)	1.43 (0.89)	*F* (2,1112) = 1.60	0.203	–
Engagement (ENG) coping strategies	1.23 (0.46)	1.17 (0.45)	1.28 (0.46)	1.24 (0.47)	*F* (2,1118) = 5.60	0.004	Sig: 1-2
Disengagement (DIS) coping strategies	1.07 (0.43)	1.05 (0.43)	1.07 (0.42)	1.10 (0.43)	*F* (2,1118) = 1.49	0.226	

*ENG, Engagement coping subscale; DIS, Disengagement coping subscale.Age group 1 (3–6-year-olds), *N* = *481*; Age group 2 (7–9- year-olds), *N* = 393; Age group 2 (10–12- year-olds), *N* = 248.Post-hoc comparisons (Bonferroni’s): only the groups between which the differences are significant are indicated.*

### Correlations Between the Study Variables

In the full sample, child’s age was positively related to favorable outcomes (e.g., reflection and social bonding) and negatively related to conduct problems and hyperactive behaviors (Table 3). An older age was also positively related to engagement coping.

**TABLE 3:** Correlation matrix and descriptive statistics of the study variables.

	1	2	3	4	5	6	7	8	9	10	11	12	13	14	15	16
1. Age																
2. SES	−0.03															
3. Close contagion	−0.02	0.06														
4. Close death	−0.04	0.01	0.29***													
5. Economic impact	0.02	−0.39***	0.04	0.04												
6. Fear of the future	−0.02	−0.24***	0.00	−0.04	0.35***											
7. Parental Resilience	−0.02	0.13***	−0.02	−0.01	−0.09*	−0.29***										
8. Engagement	0.08*	0.06*	0.01	−0.03	0.07*	0.06	0.19***									
9. Disengagement	0.05	−0.19***	0.04	0.06*	0.18***	0.35***	−0.20***	0.17***								
10. Conduct problems	−0.18***	−0.03	0.08*	0.04	0.09*	0.14***	−0.20***	−0.19***	0.27***							
11. Hyper. behaviors	−0.17***	−0.09*	0.09*	0.05	0.09*	0.17***	−0.21***	−0.13***	0.29***	0.68***						
12. Emot. problems	−0.03	−0.01	0.06*	0.03	0.04	0.13***	−0.18***	−0.09*	0.27***	0.62***	0.61***					
13. Rout. maintenance	0.03	0.08*	0.00	−0.02	−0.08*	−0.07*	0.25***	0.27***	−0.11***	−0.33***	−0.31***	−0.24***				
14. Social or. reflect.	0.22***	0.02	−0.04	0.01	0.01	0.06*	0.10*	0.34***	0.07*	−0.05	0.05	0.07*	32***			
15. Prosocial involve.	0.00	0.04	0.01	0.01	−0.01	0.05	0.21***	0.28***	0.06*	−0.17***	−0.03	−0.06*	0.32***	0.47***		
16. Social bonding	14***	0.04	−0.3	−0.02	0.00	0.03	0.10*	0.19***	0.05	−0.10*	−0.04	−0.02	0.30***	0.32***	0.32***	
Mean	7.26	0.09	–	–	1.36	1.78	2.52	1.23	1.07	2.29	2.37	2.22	2.05	2.78	2.57	2.52
SD	2.38	0.75	–	–	1.00	0.85	0,68	0.46	0.42	0.70	0.50	0.61	0.54	0.65	0.60	0.96
*N*	1123	1093	1122	1121	1120	1119	1067	1120	1120	1120	1120	1120	1075	1069	1073	1083
Range	3–12	−2.6 to 1.3	0–1	0–1	0–3	0–3	0–4	0–2.7	0–2.3	0–4	0.2–3.8	0–4	0–4	0.7–4	0–4	0–4

*p = 0.05, **p = 0.01, ***p = 0.001.

Engagement and disengagement coping were positively and modestly correlated and, as expected, indicators of adjustment and maladjustment were negatively correlated to each other.

High significant positive correlations among indicators of maladjustment (i.e., conduct problems, hyperactive behaviors and emotional problems) were found, along with moderate positive correlations with child disengagement coping. Similarly, significant positive correlations between indicators of adjustment (i.e., routine maintenance, prosocial involvement, social-oriented reflection, social bonding), with slightly lower magnitudes, were found, along with moderate positive correlations with child engagement coping.

Some of the COVID-19-related stressors (i.e., close death, economic impact, and particularly fear of the future) were positively correlated with disengagement coping. On the contrary, dispositional resilience, was negatively correlated with child disengagement and positively correlated with child engagement coping.

Finally, a higher family socioeconomic status was negatively correlated with COVID-19 perceived economic impact, self-reported parent fear of the future and child disengagement coping. Perceived high economic impact was positively and moderately correlated to self-reported parent fear of the future.

### Which Are the Associations Between Broad Dimensions of Child Coping and Behavioral, Emotional and Social Outcomes During the COVID-19 Pandemic?

Child disengagement coping was distinctively and similarly associated to negative outcomes, including both externalizing -conduct problems and hyperactive behaviors - and internalizing problems- emotional difficulties (Table 4). However, the effects of engagement and disengagement coping, yet opposites in direction, were similar in magnitude for conduct problems. Child engagement coping was distinctively and extensively associated to indicators of adjustment, and, particularly to social-oriented reflection. These associations were significant even when controlling for other covariates whose contribution is assumed to be relevant in the prediction of the variables of interest (i.e., sex, age, family SES, contextual stressors related to COVID-19 crisis and parental resilience).

**TABLE 4:** Results of multiple regression analysis showing associations between broad dimensions of children coping and behavioral, emotional and social functioning during the COVID-19 pandemic.

Predictors	Negative Outcomes						Positive outcomes							
	Conduct problems		Hyperactive behaviors		Emotional problems		Routine manteinance		Social-oriented reflection		Prosocial involvement		Social bonding	
	B (SE)	β	B (SE)	β	B (SE)	β	B (SE)	β	B (SE)	β	B (SE)	β	B (SE)	β
Sex	−0.05 (0.01)	−0.05	−0.07 (0.03)	−0.07*	0.00 (0.03)	0.00	0.09 (0.03)	0.08*	0.05 (0.04)	0.04	0.05 (0.04)	0.05	0.16 (0.06)	0.09*
Age	−0.07 (0.04)	−0.17***	−0.03 (0.01)	−0.16***	−0.01 (0.01)	−0.03	0.00 (0.01)	0.02	0.05 (0.01)	0.19***	−0.01 (0.01)	−0.02	0.05 (0.01)	0.13***
SES	0.08 (0.03)	0.08*	0.00 (0.02)	0.00	0.07 (0.03)	0.08*	−0.01 (0.03)	−0.01	0.01 (0.03)	0.01	0.03 (0.03)	0.00	0.05 (0.04)	0.04
**COVID-19 stressors**														
Close contagion	0.13 (0.06)	0.07*	0.11 (0.04)	0.08*	0.08 (0.03)	0.05	0.01 (0.04)	0.01	−0.09 (0.05)	−0.05	0.00 (0.05)	0.00	−0.10 (0.08)	−0.04
Close death	−0.06 (0.01)	−0.02	0.01 (0.08)	0.00	−0.04 (0.10)	−0.01	−0.02 (0.08)	−0.01	0.14 (0.09)	0.05	0.08 (0.08)	0.03	−0.01 (0.14)	0.00
Economic impact	0.05 (0.02)	0.08*	0.02 (0.02)	0.03	0.01 (0.02)	0.02	−0.05 (0.02)	−0.09*	−0.02 (0.02)	−0.03	−0.03 (0.02)	−0.04	−0.01 (0.03)	−0.01
Fear of the future	0.01 (0.03)	0.02	0.02 (0.02)	0.03	0.03 (0.02)	0.04	0.03 (0.02)	0.05	0.07 (0.03)	0.09*	0.06 (0.02)	0.09*	0.07 (0.04)	0.06
**Parent resilience**														
Trait resilience	−0.09 (0.03)	−0.09*	−0.08 (0.02)	−0.11***	−0.08 (0.03)	−0.09*	0.16 (0.03)	0.20***	0.07 (0.03)	0.07*	0.16 (0.03)	0.18***	0.11 (0.05)	0.06*
**Child coping**														
Engagement	−0.34 (0.05)	−0.22***	−0.16 (0.03)	−0.14***	−0.17 (0.04)	−0.12***	0.29 (0.04)	0.25***	0.44 (0.04)	0.31***	0.31 (0.04)	0.24***	0.32 (0.07)	0.15***
Disengagement	0.47 (0.05)	0.28***	0.33 (0.04)	0.28***	0.39 (0.04)	0.28***	−0.15 (0.03)	−0.12***	0.00 (0.05)	0.00	0.05 (0.05)	0.04	0.08 (0.08)	0.04
*R*^2^		0.20***		0.18***		0.12*		0.16***		0.15***		0.11*		0.8*

^∗^p < 0.05, **p < 0.01, ***p < 0.001. These results are derived from the total sample. They were replicated by age group subsamples in complementary analysis (3–6-year-olds, 7–9- year-olds and 10–12- year-olds). We modeled pathways from all covariates (age, sex, and SES) to all other predictors in the regression models. Main predictors were mean centered before entered into the equation.

Additionally, as regards of the main effects of the contextual factors, the existence of a close contagion and a higher parent-perceived economic impact of the COVID-19 crisis on family, were positively and significantly associated to higher levels of externalizing behaviors such as conduct problems and hyperactive behaviors. On the contrary, higher levels of parent self-reported fear of the future were positively and significantly associated to adaptative outcomes such as higher levels of social-oriented reflection and prosocial involvement. Similarly, parental resilience was positively related to positive outcomes across all the models exploring adjustment, particularly to children’s routine maintenance and prosocial involvement with others during the COVID-19 crisis.

### Which Are the Contextual Characteristics Interacting With Child Coping to Predict Outcomes During the COVID-19 Pandemic?

On the basis of the results previously obtained, after having modeled the main effects of child coping on different indicators of adjustment, we created interaction terms between child disengagement coping and contextual factors to specifically predict negative outcomes, and engagement coping and contextual factors to specifically predict positive outcomes (see Supplementary Table 2).

**FIGURE 1:**
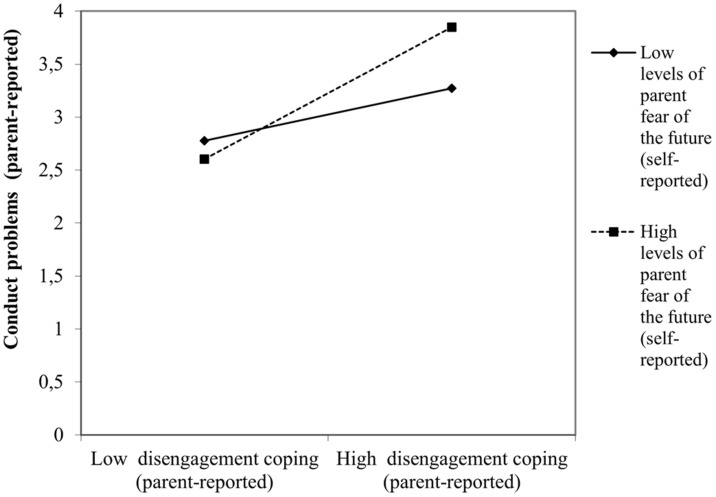
Interactive effect of parent self-reported fear of the future with child disengagement coping to predict child maladjustment concurrent to the COVID-19 pandemic: conduct problems.

**FIGURE 2:**
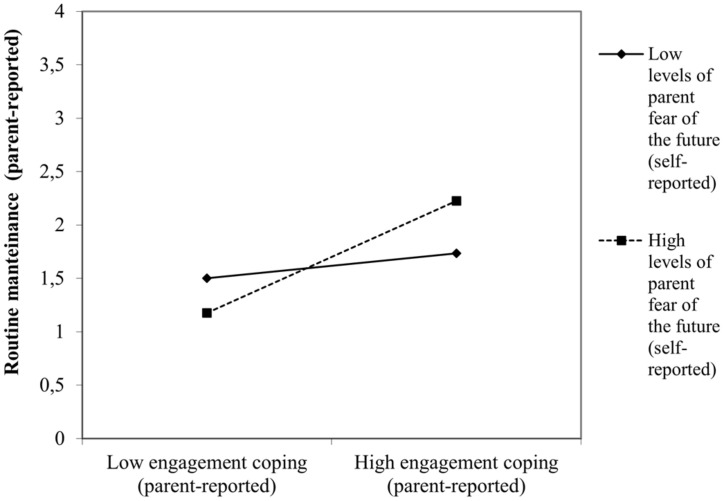
Interactive effect of parent self-reported fear of the future with child engagement coping to predict child adjustment concurrent to the COVID-19 pandemic: routine maintenance.

When interaction terms were introduced in the regression models, most of the situational stressors analyzed did not interact with children coping to predict either maladjustment or adjustment. Only parent perceived fear of the future interacted with child disengagement and engagement coping tendencies to predict negative and positive consequences, respectively. For instance, higher levels of parent perceived fear of the future predicted higher levels of child behavioral (β = 0.13, *p* < 0.04, Figure 1) and emotional problems (β = 0.14, *p* < 0.03) when children displayed disengagement coping. On the contrary, higher levels of parent perceived fear of the future predicted higher levels of child social-oriented reflection (β = 0.17, *p* < 0.03) when the children coping style was engaged-oriented (Figure 2).

### Does Parental Dispositional Resilience Interact With Child Broad Dimensions of Coping to Predict Outcomes During the COVID-19 Pandemic?

Similar to what was done for other contextual factors and based on the results previously obtained, we created interaction terms between child disengagement and engagement coping with parental resilience to specifically, and respectively, predict negative and positive outcomes (see Supplementary Table 3).

**FIGURE 3:**
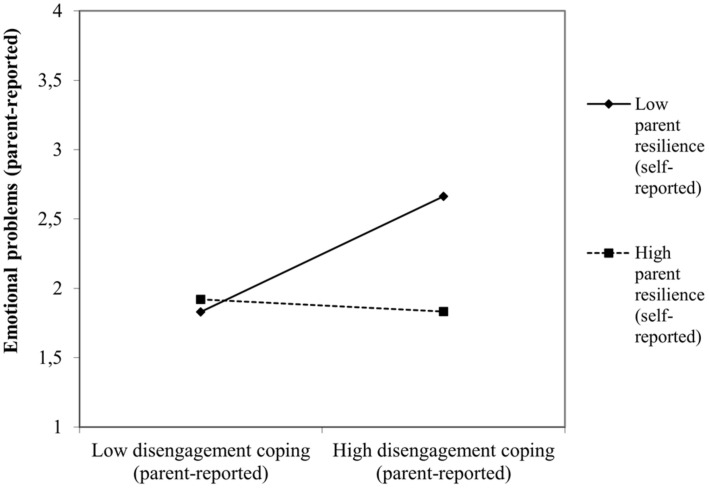
Interactive effect of self-reported parent resilience with child disengagement coping to predict child maladjustment concurrent to the COVID-19 pandemic: emotional problems.

**FIGURE 4:**
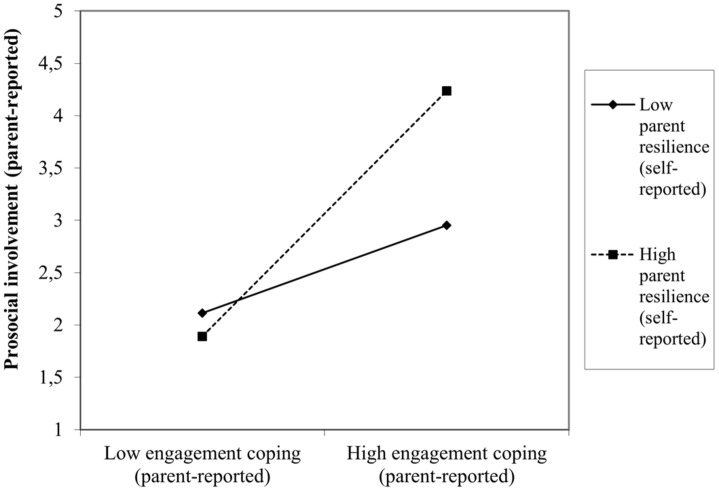
Interactive effect of self-reported parent resilience with child disengagement coping to predict child adjustment concurrent to the COVID-19 pandemic: prosocial involvement.

There was an interactive effect of parental dispositional resilience with child situational coping for the prediction of both negative and positive outcomes resulting from the COVID-19 crisis. Specifically, lower levels of parent resilience interact with high levels of child disengagement coping to produce higher levels of emotional problems (β = −0.07, *p* < 0.02, Figure 3). Conversely, higher levels of parent resilience enhance the child prosocial attitude toward others during the COVID-19 crisis when they tend to use approach-oriented coping strategies (β = 0.10, *p* < 0.001, Figure 4).

### Are the Main and Interactive Effects Found Age-Dependent?

To test if the main and interactive effects of interest varied in function of age, we run the same previous models by age group subsamples in complementary analysis, addressing the preschool period, middle childhood and early adolescence (see Supplementary Tables 4, 5). Overall, the findings pertaining the main effects of coping remained unaltered, but some age-determined differences emerged when analyzing the contextual interactions by age-group.

First, for all three age groups, disengagement coping strategies predicted higher levels of conduct problems, hyperactive behaviors and emotional problems (Supplementary Table 4). On the contrary, for all the three age groups, engagement coping was associated to a better behavioral and psychosocial adjustment (routine maintenance, prosocial involvement, social-oriented reflection and social bonding (Supplementary Table 5).

However, the moderator effects of contextual stressors on the child coping-outcomes relation were slightly different in function of the age group considered to predict both negative and positive outcomes. Only preschoolers, but not older children (i.e., above 7 years of age) were particularly vulnerable to experience behavioral (β = 0.21, *p* < 0.02) and emotional difficulties (β = 0.22, *p* < 0.01) when displaying a disengagement coping style in the context of a higher parent self-reported fear of the future. At the same time higher parent self-reported fear of the future also positively influenced the routine maintenance (β = 0.23, *p* < 0.02) of young engaged-oriented children but not their older counterparts (i.e., above 9-year-olds). For 7–9-year-old children, however, the existence of a COVID-19 contagion in the close social circle was the stressor whose interaction with a disengagement coping was significantly associated to a poorer emotional functioning (β = 0.11, *p* < 0.05). Finally, for early adolescents (i.e., 10–12-year-olds) none of the situational stressors analyzed interacted with disengagement or engagement tendencies to produce particular outcomes.

Similarly, the moderator effects of the parental dispositional resilience on the child coping-outcomes relation were slightly different by age group subsamples, fundamentally when predicting negative outcomes. Only preschoolers seemed particularly vulnerable to the effect of low levels of parent resilience. Specifically, lower levels of parent self-reported resilience interacted with high levels of disengagement coping to produce higher levels of conduct problems (β = −0.08, *p* < 0.02), hyperactive behaviors (β = −0.09, *p* < 0.007) and emotional problems (β = −0.12, *p* < 0.001) in children aged 3–6. Interestingly, for 7–9-year-old children, lower levels of parent-self reported resilience interact with high levels of child engagement coping to predict higher levels of child social-oriented reflection (β = −0.13, *p* < 0.006).

## Discussion

Our main goal was to examine how child coping, unique contextual conditions and parent resilience, might contribute to children’s psychosocial adjustment during the COVID-19 pandemic, incorporating in our analysis a developmental perspective. Consistent with our hypothesis, the psychological impact of the pandemic on children well-being, might be variable, depending on individual and situational characteristics, which would serve, independently and through person by context interactions, as risk or protective factors for children adjustment to these circumstances.

We first studied the main effects of child context-specific coping on psychosocial well-being. Interestingly, our findings show that, disengagement coping is associated to negative outcomes, whereas engagement coping would be predictive of concurrent child psychosocial adjustment during the pandemic. Contrary to what would be expected due to the uncontrollable nature of the stressful situation, child disengagement coping distinctively accounted for negative outcomes, both externalizing and internalizing, and this pattern of findings was replicated in all of three age levels in complementary age-group analysis, suggesting that this association might emerge early in preschool years and remain significant across early development. Thus, our results are in line with other findings from preschool samples examining normative uncontrollable developmental stressors (e.g., night fears, Chalmers et al., 2011), but clearly diverge from the main line of findings pertaining youth populations, testing the effects of other normative uncontrollable stressors (e.g., medical procedures, parental conflict, Band and Weisz, 1988; Altshuler and Ruble, 1989; O’Brien et al., 1995). In adult literature, however, there is evidence suggesting that avoidant coping in the context of a pandemic (e.g., SARS) is predictive of higher levels of psychological symptoms (Main et al., 2011). Unsurprisingly, certain tendency of younger children to rely more prominently on disengagement coping strategies during the current pandemic would be expected. Similarly, the use of these type of strategies by children whose emerging metacognitive capacities allow them to distinguish between controllable and uncontrollable situations (i.e., children about 7 years old, Altshuler and Ruble, 1989). However, to explain their negative effect on child well-being it might be essential to recall some of their specific functions. Just as some of the disengagement tactics could mitigate the short-term impact of the uncontrollable COVID-19-related stressors (e.g., cognitive and behavioral distraction allowing the redirection of attention from the stressor to an alternative target) others (e.g., partial or complete avoidance), might prevent children from detecting, appraising and dealing with the current crisis in more potential adaptive ways (e.g., reminding him/herself that his/her situation is not that bad), exacerbating the behavioral and emotional negative consequences of the stressors (Compas et al., 2001; Skinner and Zimmer-Gembeck, 2016c).

As expected, we found a moderator effect of contextual factors on the relation between child coping and adjustment. However, remarkably, this effect was only significant for parent self-reported fear of the future and not any of the other contextual factors examined (i.e., close COVID-19 contagion and/or death, and perceived economic impact of the health crisis). This finding is subjected to different, yet complementary, interpretations. First, rather than solely by the experiencing of “objective stressors” in their close social circle, children’s coping and adjustment processes seem to be more dependent on “subjective-like factors” exerting their influence from the proximity of their *immediate* socialization circle, in a developmental period in which, contrarily to others (e.g., adolescence) parental influences on child coping responses are more prominent (Kliewer et al., 1996; Skinner and Zimmer-Gembeck, 2016b). Second, the moderator effect of these stressors could be only properly examined if the potential confounding effect of children’s knowledge about the circumstances (e.g., does she/he know about the close contagion?) was controlled in our study. It might be the case that these pandemic-related stressors do not have a significant effect on child engagement and disengagement coping strategies to produce specific outcomes, simply due to the absence of children’s explicit knowledge about them. In any case, our findings suggest that high levels of parental fear of the future do have a moderator effect on child coping-outcomes relation. In fact, this effect might be described as paradoxical, as higher levels of parental fear of the future could serve as both a risk or protective factor for child psychosocial well-being, depending on which type of child coping they interact with (disengagement and engagement, respectively). This might be partially explained by the sense of threat and the uncertainty that COVID- 19 pandemic has caused (Peters et al., 2017). Despite we have adaptive mechanisms that allow us to successfully navigate the inherent uncertainties of life (e.g., the reliance on past experiences when trying to anticipate future events, Grupe and Nitschke, 2013), they are very likely to be insufficient and their beneficial effects limited, when we must function under highly stressful situations such as the current COVID-19 pandemic (Wang et al., 2020). In these conditions, we are forcedly and abruptly confronted with high levels of uncertainty about a seemingly uncontrollable and unpredictable imminent future (Pfefferbaum and North, 2020). Because of this uncertainty, even as diffuse or tangible as the threat might be (Baker et al., 2020), our anxiety levels could increase because our perceived capacity of anticipation is diminished (Grupe and Nitschke, 2013). Thus, in this scenario, parent fear of the future would act as an amplifier of the child disengagement coping negative effect on child emotional and behavioral adjustment. Yet, at the same time, and interactively with child engagement coping, it could exert a protective effect, by functioning as a trigger to initiate compensatory processes aimed to restore a certain sense of predictability and controllability, through simple actions directed to maintain daily routines during the pandemic home-confinement. Essentially, these findings are explicitly confirming not only the importance of contextual risk (Main et al., 2011), but also the important role of the individual differences in coping. Ultimately, the consequences of high levels of the same contextual stressor (i.e., fear of the future) depend on the type of coping on which they operate.

Similarly, and supporting our hypothesis, parental resilience also exerts a moderator effect on child coping-outcomes relation. In fact, our findings suggest that both risk and resilience to maladjustment can result from these interactive processes. The parental personality trait of resistance to adversity (i.e., resilience, Bensimon, 2012) interacts with child engagement and disengagement coping to produce positive or negative outcomes. First, low levels of parent resilience could serve as a risk factor for emotional difficulties at high, but not low levels of child disengagement coping. Therefore, consistent with classical hypothesis, our results suggest that this differential effect of parental resilience seems uniquely determinant in the presence of high, but not low levels of risk (Stattin et al., 1997). Second, a similar, yet less strong effect, is found when parental resilience operates along with child engagement tendencies, as its positive effect is clearly strong for higher levels of child engagement coping compared to lower levels. Interestingly, high levels of engagement and resilience coming together or, conversely, high levels of disengagement and low of resilience combined, might be the reflection of the underneath similarities between child and parent coping resulting from socialization processes (Kliewer et al., 1996) which remain on course during the pandemic. Thus, a parental dispositional adaptive coping would be essential to explain not only the concurrent level of child adjustment to the COVID-19 crisis, but also to understand how children cope the way they do under these conditions (Abaied et al., 2010; Cappa et al., 2011; Skinner and Zimmer-Gembeck, 2016b). In fact, despite being an atypical and non-normative scenario, the pandemic is still an opportunity for parents and children to advance in the coping socialization tasks.

Altogether, these findings reinforce, on the basis of a systemic theory, the interdependence principle, as the functioning of one family member impacts the others and vice versa (Carr, 2015). Moreover, systems theory could serve as a useful framework to integrate these findings, by signaling concrete channels (e.g., parent resilience) through which broader contextual risk (e.g., economic pressure on especially vulnerable families) negatively affect individual family members (e.g., child adjustment, Prime et al., 2020).

Finally, as hypothesized, interesting age-group differences emerged in our complementary analysis. As coping is both a reflection and a contribution to development (Zimmer-Gembeck and Skinner, 2011b), children age influences not only the distinctive strategies they use, but also the vulnerabilities that might lead them to short-term negative outcomes within the context of the pandemic. Undoubtedly, COVID-19 crisis has immersed children into an environment of unprecedent challenging demands, likely to be particularly overwhelming for younger ones (Fegert et al., 2020; Green, 2020). Hence, as our findings show, the higher vulnerability of preschoolers to distal and proximal contextual risk is not surprising. For instance, consistent with the idea that the parent-child interpersonal coping systems are initially coregulated, it is reasonable that young children depend on external sources (e.g., parent trait resilience) for regulation (Skinner and Zimmer-Gembeck, 2007). Conversely, as our findings suggest, older children’ permeability to the influence of COVID-19 related stressors could be lower in favor of a prominent role of their individual resources (i.e., coping skills), as a consequence of their behavioral and cognitive coping skills becoming more diverse and complex in nature as they grow.

Considering the current predictions about likely recurrent future COVID-19 breakouts and the lack of any previous similar experiences in our recent history, our findings might provide some insights to guide, at a practical level, the identification of individual and contextual risk, informing tailored preventive interventions aimed to reduce the psychosocial impact of future pandemic recurrences on children of different ages. Certainly, coping-based interventions are inherently a difficult endeavor (Coyne and Racioppo, 2000) and children coping-based interventions might be subjected to additional challenges (Frydenberg et al., 2017). However, there is room for hope. Universal stress management programs have shown positive outcomes for children (e.g., reduced levels of stress and anxiety, Kraag et al., 2009) and schools seem to be feasible settings for their application (Pincus and Friedman, 2004). Moreover, besides children, the beneficial effects of these programs are extensible to their parents (Frydenberg et al., 2014). Consequently, any intent to meaningfully adapt and transfer some of these effective preventive intervention’s components to the highly specific context of the COVID-19 pandemic, would be an interesting contribution to the field of children coping research and more importantly, a mighty useful service to children and families during the COVID-19 times.

### Strengths and Limitations

To our knowledge, this is one of the first studies to date, to analyze the coping-outcomes relation within the context of the COVID-19 pandemic. Despite examining the changes derived from the crisis on a large community sample of Spanish home-confined children during the acute phase of the pandemic, using a wide range of adjustment measures, and incorporating a developmentally friendly approach, this research has important methodological limitations.

First, the measures for the coping construct may be limited. As a result of prioritizing a shorten length for the coping measure due to time-cost reasons, the number of items selected might fail to capture the wide variety of strategies used by children of these ages. Also, despite following a theory driven strategy in the *ad hoc* development of the questionnaire and considering the adequacy of the items to the current crisis for their selection, this procedure has clear limitations. For instance, the content of some items is likely to be age un-appropriated or too abstract to be applicable to young children. For this reasons, empirical difficulties to reach acceptable fit indices in factor/confirmatory analysis and, also, scale-reliability analysis would be expected. Second, some of the subscales used to operationalize child adjustment during the pandemic showed unacceptable levels of internal consistency (e.g., routine maintenance, α = 0.55; social bonding, α = 0.48). This could be partially explained, in the latter case, by the small number of items composing the scale (i.e., 2 items). Third, data collection relied exclusively on parent reports of observable and non-observable child behavior. Besides the threat of single informant biases, without a multi-informant approach, we lack valuable self-report information, particularly interesting in the case of older children (e.g., early adolescents). Fourth, cross-sectional analyses are only informative of short-term effects, offering a limited view on the true scope and magnitude of the pandemic impact on children. To test if these effects are sustained over time, longitudinal analysis to compare them during versus after quarantine would be needed. Additionally, without a prospective design it is not possible to make causal inferences on coping-outcomes relation or explore possible reciprocal effects. Fifth, relevant pre-COVID-19 child and family predictors accounting for child functioning during the current crisis were not included in our analysis (e.g., serious family economic hardships). Sixth, the cumulative effects of concurrent additional stressors to the COVID-19 crisis were not modeled (e.g., chronic child health condition or domestic violence). Finally, we did not control the presumably important effect of the moment, that is, the specific date, when the data was collected (i.e., at the beginning or the end of the home confinement period).

With more sophisticated and rigorous designs, combining multi-informant and multi-method assessments with a longitudinal approach, future research should necessary address the specific emotional, behavioral and cognitive mechanisms through which children coping during extreme circumstances such the COVID-19 pandemic, exerts its influence to produce specific outcomes. Specifically, a longitudinal follow-up of our study sample, would provide a better picture of how children cope with the long-standing pandemic-related stressors beyond the acute phase examined for this work, providing valuable insights on the mechanisms involved in potential maladaptive courses observed in children with particularly higher levels of vulnerability due to cumulative risk.

### Conclusions

Our findings contribute to better understand how children adapt (or fail to) during the COVID-19 pandemic by highlighting the explanatory value of child context-specific coping, pandemic and family contextual factors and child development level over observed adjustment. Overall, they suggest the need of combining both child and family components in tailored-preventive interventions aimed to reduce the psychological impact of future pandemic outbreaks, as how children and their parents cope plays a crucial role for their adjustment. Also, they confirm the need to adopt a developmentally sensitive perspective in which aged-graded specifications are considered.

## Data Availability Statement

The datasets presented in this article are not readily available in a public repository. However, they are expected to be publicly available soon. Until then, they will be available upon request to the corresponding author. Requests to access the datasets should be directed to BD-Á, beatrizdominguez.alvarez@usc.es.

## Ethics Statement

The studies involving human participants were reviewed and approved by the Bioethics Committee of the Universidade de Santiago de Compostela. Written informed consent to participate in this study was provided by the participants’ legal guardian/next of kin.

## Author Contributions

All authors were involved in the process of data collection. LL-R, JG-F, AI-T, and ER organized and prepared the database for the statistical analysis. BD-Á and JG-F contributed to the conception and design of the study. BD-Á performed the statistical analysis and wrote the manuscript. All authors contributed to the revision of the manuscript and read and approved the submitted version.

## Conflict of Interest

The authors declare that the research was conducted in the absence of any commercial or financial relationships that could be construed as a potential conflict of interest.
