# Supplementary material for: Children Coping, Contextual Risk and Their Interplay During the COVID-19 Pandemic: A Spanish Case
Source: Front Psychol. 2020 Dec 16;11:577763. doi: 10.3389/fpsyg.2020.577763 (PMC7772313; doi:10.3389/fpsyg.2020.577763)
Supplement: Supplementary file 1 [file Table_1.docx]

Supplementary Material

**Table 1S.** Items of the child coping measure used in the CONFIA-20 study, specific coping strategies assessed and original coping measures from which they were selected

| **Broad dimension of coping: engagement** | **Specific coping strategy** | **Original coping measure** |
| --- | --- | --- |
| 2.Tries to hold a positive view of the situation | Cognitive restructuring | KidCOPE |
| 5.Spontaneously proposes possible solutions to current crisis | Problem solving | KidCOPE |
| 8.Spends time with other people (e.g. family members) to feel better | Social support | KidCOPE |
| 11.Tries to do specific actions to solve the current crisis | Problem solving | KidCOPE |
| 12.Tries to calm him/herself | (Positive) Emotion regulation | KidCOPE |
| 15.Shares with us how she/he feels regarding the crisis | Emotional support seeking | CCSC-R1 |
| 16.Tries to understand how things like this happens | Seeking understanding | CCSC-R1 |
| 17.Makes jokes or tries to laugh about the current situation | Humor | Ad hoc item |
| 18.Seeks help in others to understand what is happening | Information-driven support seeking | Ad hoc item |
| 19.Reminds him/herself that, globally, his/her situation is not that bad | Positivity | CCSC-R1 |
| 21.Seeks help to try to improve the situation | Instrumental-driven support seeking | Ad hoc item |
| **Broad dimension of coping: disengagement** |  |  |
| 1.Seems to try to forget what is happening | Distraction | KidCOPE |
| 3.Prefers to spend time alone | Social withdrawal | KidCOPE |
| 4.Blames someone for causing the current crisis | Blaming others | KidCOPE |
| 6.Yells or gets angry | (Negative) Emotion regulation | KidCOPE |
| 7.Wishes the COVID-19 crisis had never happened | Wishful thinking | KidCOPE |
| 9.Does things (e.g. play or watch TV) to evade him/herself | Distraction | KidCOPE |
| 10.Avoids talking about the current situation | Avoidance | CCSC-R1 |
| 13.Wishes something could be done to change the current situation | Wishful thinking | KidCOPE |
| 14.Remains without doing nothing because thinks that the situation can’t be solved) | Resignation | KidCOPE |
| 20.Avoids thinking about the current crisis | Avoidance | CCSC-R1 |
| 22.Fantasizes with a prompt resolution for this situation | Wishful thinking | CCSC-R1 |
| ***Note:*** KidCOPE (Spirito, 1988)  CCSC-R1 (Children’s Coping Strategies Checklist, Ayers et al., 1996) |  |  |

**Table 2S.** Results of multiple regression analysis showing the interactive effect of COVID-19-related stressors with broad dimensions of children coping to predict behavioral, emotional and social functioning during the pandemic

| **Covariates** | **Negative Outcomes** | | | | | | **Positive outcomes** | | | | | | | | |
| --- | --- | --- | --- | --- | --- | --- | --- | --- | --- | --- | --- | --- | --- | --- | --- |
|  | **Conduct problems** | | **Hyperactive**  **behaviors** | | **Emotional problems** | | **Routine manteinance** | | **Social-oriented reflection** | | **Prosocial**  **involvement** | | **Social bonding** | | |
|  | **B (SE)** | **β** | **B (SE)** | **β** | **B (SE)** | **β** | **B (SE)** | **β** | **B (SE)** | **β** | **B (SE)** | **β** | **B (SE)** | **β** |  |
| Sex | -.07 (.04) | -.06* | -.08 (.03) | -.08* | .00 (.03) | .00 | .09 (.03) | .09* | .05 (.04) | .04 | .06 (.03) | .05 | .16 (.06) | .08* |  |
| Age | -.06 (.01) | -.19*** | -.04 (.06) | -.17*** | -.01 (.03) | -.04 | .00 (.01) | .01 | .05 (.01) | .19*** | .00 (.01) | -.01 | .05 (.01) | .13*** |  |
| SES | .05 (.03) | .06 | .00 (.02) | .00 | .06 (.03) | .07* | .00 (.03) | .00 | .01 (.03) | .01 | .00 (.03) | .00 | .04 (.04) | .03 |  |
| **COVID-19 stressors** | | | | | | |  | | | | | | | | |
| Close contagion | .13 (.06) | .07* | .11 (.04) | .08* | .08 (.05) | .05 | .01 (.04) | .01 | -.08 (.05) | -.05 | .00 (.05) | .00 | -.10 (.08) | -.04 |  |
| Close death | -.02 (.10) | -00 | .01 (.08) | .01 | -.01 (.10) | -.01 | -.05 (.02) | -.02 | .14 (.09) | .05 | .08 (.08) | .03 | .00 (.14) | .00 |  |
| Economic impact | .04 (.02) | .06 | .01 (.02) | .02 | .00 (.02) | .00 | -.05 (.02) | -.09* | -.02 (.02) | -.03 | -.02 (.02) | -.04 | -.01 (.03) | -.01 |  |
| Fear of the future | .00 (.03) | .00 | .01 (.02) | .02 | .03 (.02) | .04 | .01 (.02) | .02 | .07 (.02) | .09* | .07 (.02) | .10* | .09 (.04) | .08* |  |
| **Parent resilience** | | | | | | |  | | | | | | | | |
| Trait resilience | -.14 (.03) | -.14* | -.10 (.02) | -.14*** | -.10 (.03) | -.12*** | .16 (.03) | .20*** | .07 (.03) | 07* | .15 (.03) | .18*** | .11 (.05) | .08* |  |
| **Child coping** | | | | | | |  | | | | | | | | |
| Disengagement | .22 (.11) | .14* | .23 (.07) | .20*** | .19 (.08) | .14* |  |  |  |  |  |  |  |  |  |
| Engagement |  |  |  |  |  |  | .08 (.08) | .07 | .34 (.10) | .24*** | .18 (.09) | .14* | .21 (.15) | .10 |  |
| **Contextual interactions** | | | | | | |  | | | | | | | | |
| Dis x Cont | .06 (.13) | .01 | -.03 (.09) | -.01 | .05 (.12) | .01 |  |  |  |  |  |  |  |  |  |
| Dis x Deat | -.26 (.27) | -.03 | .01 (.23) | .00 | -.26 (.29) | -.03 |  |  |  |  |  |  |  |  |  |
| Dis x Econ | .00 (.05) | .01 | -.01 (.03) | -.02 | .00 (.04) | .00 |  |  |  |  |  |  |  |  |  |
| Dis x Fear | .11 (.06) | .13* | .05 (.04) | .09 | .10 (.05) | .14* |  |  |  |  |  |  |  |  |  |
| Eng x Cont |  |  |  |  |  |  | -.11 (.10) | -.04 | -.15 (.12) | -.04 | -.22 (.12) | -.07 | .08 (.20) | .02 |  |
| Eng x Deat |  |  |  |  |  |  | .07 (.16) | .01 | .17 (.17) | .03 | .14 (.19) | .24 | .06 (.35) | .00 |  |
| Eng x Econ |  |  |  |  |  |  | .02 (.04) | .03 | .00 (.04) | -.01 | .00 (.04) | .00 | .07 (.07) | .06 |  |
| Eng x Fear |  |  |  |  |  |  | .10 (.05) | .17* | .00 (.04) | .09 | .10 (.05) | .15 | .01 (.08) | .01 |  |
| **R^2^** | .10*** | | .12*** | | .06*** | | .10*** | | .14*** | | .09*** | | .06* | | |
| ***Note****.* *p<.05, **p<.01, ***p<.001.  These results are derived from the total sample. Complementary analysis by age group subsamples were run to identify specific coping x context interactions for each group of aged children  We modeled pathways from all covariates (age, sex, and SES) to all other predictors in the regression models. Main predictors were mean centered before entering into the equation. Interaction terms were created from centered values. | | | | | | | | | | | | | | | |

**Table 3S.** Results of multiple regression analysis showing the interactive effect of parental coping with broad dimensions of children coping to predict behavioral, emotional and social functioning during the COVID-19 pandemic

| **Covariates** | **Negative Outcomes** | | | | | | **Positive outcomes** | | | | | | | | |
| --- | --- | --- | --- | --- | --- | --- | --- | --- | --- | --- | --- | --- | --- | --- | --- |
|  | **Conduct**  **Problems** | | **Hyperactive Behaviors** | | **Emotional**  **Problems** | | **Routine**  **Manteinance** | | **Social -oriented reflection** | | **Prosocial**  **involvement** | | **Social bonding** | | |
|  | **B (SE)** | **β** | **B (SE)** | **β** | **B (SE)** | **β** | **B (SE)** | **β** | **B (SE)** | **β** | **B (SE)** | **β** | **B (SE)** | **β** |  |
| Sex | -.07 (.04) | -.05 | -.07 (.03) | -.08* | .00 (.03) | .00 | .09 (.03) | .09* | .04 (.04) | .04 | .06 (.03) | .05 | .16 (.06) | .09* |  |
| Age | -.06 (.01) | -.19*** | -.04 (.01) | -.17*** | -.01 (.01) | -.04 | .00 (.01) | .01 | .05 (.01) | .19*** | -.01 (.01) | -.01 | . 05 (.01) | .13*** |  |
| SES | .06 (.03) | .04 | -.01 (.02) | -.01 | .06 (.03) | .07* | .00 (.03) | .00 | .01 (.03) | .01 | -.01 (.03) | -.01 | .04 (.04) | .03 |  |
| **COVID-19 stressors** | | | | | | |  | | | | | | | | |
| Close contagion | .13 (.06) | .07* | .11 (.04) | .08* | .08 (.05) | .05 | .01 (.04) | .01 | -.09 (.05) | -.05 | .00 (.05) | .00 | -.10 (.08) | -.04 |  |
| Close death | -.04 (.10) | -.01 | .02 (.09) | .01 | -.03 (.10) | -.01 | -.04 (.08) | -.01 | .14 (.09) | .05 | .09 (.08) | .03 | .00 (.14) | .00 |  |
| Economic impact | .04 (.02) | .06 | .01 (.02) | .02 | .01 (.02) | .01 | -.05 (.02) | -.09* | -.02 (.02) | -.03 | -.03 (.02) | -.05 | -.01 (.03) | -.01 |  |
| Fear of the future | .00 (.03) | .00 | .01 (.02) | .02 | .03 (.02) | .04 | .01 (.02) | .02 | .07 (.02) | .09* | .07 (.02) | .10* | .08 (.04) | .08* |  |
| **Parent resilience** | | | | | | |  | | | | | | | | |
| Trait resilience | -.15 (.03) | -.14*** | -.11 (.02) | -.15*** | -.13 (.06) | -.13*** | .17 (.03) | .21*** | .07 (.03) | .07* | .15 (.03) | .17*** | .11 (.05) | .08* |  |
| **Child coping** | | | | | | |  | | | | | | | | |
| Disengagement | .40 (.05) | .25*** | .30 (.03) | .26*** | .37 (.04) | .26*** |  |  |  |  |  |  |  |  |  |
| Engagement |  |  |  |  |  |  | .27 (.04) | .23*** | .44 (.04) | .31*** | .31 (.04) | .24*** | .33 (.07) | .16*** |  |
| **Coping interaction** | | | | | | |  | | | | | | | | |
| Dis x Res | -.05 (.07) | -.02 | -.07 (.05) | -.05 | -13 (.06) | -.07* |  |  |  |  |  |  |  |  |  |
| Eng x Res |  |  |  |  |  |  | .08 (.07) | .04 | .02 (.06) | .01 | .18 (.06) | .10* | .10 (.10) | .03 |  |
| **R^2^** | .12*** | | .14*** | | .09*** | | .12*** | | .16*** | | .12*** | | .07*** | | |
| ***Note****.* *p<.05, **p<.01, ***p<.001.  These results are derived from the total sample. Complementary analysis by age group subsamples were run to identify specific coping x coping interactions for each group of aged children  We modeled pathways from all covariates (age, sex, and SES) to all other predictors in the regression models. Main predictors were mean centered before entering into the equation. Interaction terms were created from centered values. | | | | | | | | | | | | | | | |

**Table 4S.** Results of multiple regression analysis showing main and interactive effects of broad dimensions of children coping, contextual risk and parent resilience to predict behavioral, emotional and social functioning during the COVID-19 pandemic: negative outcomes by age groups

| **Covariates** |  | |  | **Negative Outcomes** | | | | | |  |  |
| --- | --- | --- | --- | --- | --- | --- | --- | --- | --- | --- | --- |
|  | **Conduct problems** | | | | **Hyperactive behaviors** | | | **Emotional problems** | | |  |
|  | **Age 1** | **Age 2** | | **Age 3** | **Age 1** | **Age 2** | **Age 3** | **Age 1** | **Age 2** | **Age 3** |  |
|  | **β** | **β** | | **β** | **β** | **β** | **β** | **β** | **β** | **β** |  |
| Sex | -.13* | .02 | | .00 | -.11* | -.02 | -.09 | -.03 | .00 | -.03 |  |
| Age | -.08* | -.09* | | -.06 | -.08 | -.07. | -.11 | .01 | -.02 | -.12 |  |
| SES | .12* | .08 | | -.02 | .02 | .01 | -.01 | .11* | .12* | .00 |  |
| ***Main effects*** | | | | | | | | | | |  |
| **COVID-19 stressors** | | | | | | | | | | |  |
| Close contagion | .11* | .05 | | .00 | .09* | .12* | -.04 | .05 | .09 | -.02 |  |
| Close death | .00 | -.07 | | .06 | .01 | -.05 | .06 | .02 | -.08 | .02 |  |
| Economic impact | .05 | .14* | | .03 | -.04 | .10 | .10 | .00 | .02 | .04 |  |
| Fear of the future | .06 | -.08 | | .01 | .11* | -.02 | -.07 | .13* | -.04 | .00 |  |
| **Parent resilience** | | | | | | | | | | |  |
| Trait resilience | -.02 | -.12* | | -.21* | -.07 | -.07 | -.29*** | -.09 | -.01 | -.21* |  |
| **Child coping** | | | | | | | | | | |  |
| Disengagement | .32*** | .30*** | | .19* | .30*** | .31*** | .28*** | .24*** | .31*** | .32*** |  |
| Engagement | -.28*** | -.22*** | | -.10 | -.24*** | -.14* | -.04 | -.20*** | -.13* | -.04 |  |
| **R^2^** | .22*** | .19*** | | .11 | .20*** | .16*** | .21*** | .13*** | .14* | .17*** |  |
| ***Interactive effects* (I)** | | | | | | | | | | | |
| **Contextual interaction** | | | | | | | | | | |  |
| Diseng x Contagion | .01 | .02 | | .01 | -.02 | .03 | .01 | .00 | .11* | -.08 |  |
| Diseng x Death | -.05 | .00 | | -10 | -.03 | -.04 | .01 | -.05 | -.04 | -.08 |  |
| Diseng x Econ. impact | -.09 | .17 | | -.02 | -.07 | .06 | .06 | -.09 | .11 | .10 |  |
| Diseng x Fear of the future | .21* | -.02 | | .04 | .07 | .11 | -.05 | .22* | -.01 | .02 |  |
| **R^2^** | .12* | .13* | | .17* | 13* | .09* | .20* | .10* | .09* | .17* |  |
| ***Interactive effects* (II)** | | | | | | | | | | |  |
| **Coping interaction** | | | | | | | | | | |  |
| Dis x Res | -.08* | -.04 | | -.05 | -.09* | -.10* | -.08 | -.12* | -.04* | -.03 |  |
| **R^2^** | .12*** | .14*** | | .11* | 13*** | .14*** | .22*** | .08*** | .11* | .18*** |  |
| ***Note.*** *p<.05, **p<.01, ***p<.001.  Age 1= group of 3-6-year-olds, N= 481; Age 2=group 2 of 7-9- year-olds, N= 393; Age 3: group of 10-12- year-olds, N=248. | | | | | | | | | | |  |

**Table 5S.** Results of multiple regression analysis showing main and interactive effects of broad dimensions of children coping, contextual risk and parent resilience to predict behavioral, emotional and social functioning during the COVID-19 pandemic: positive outcomes by age groups

| **Covariates** | | |  |  | | | **Positive Outcomes** | | | | | | | |  | |  |  |  |
| --- | --- | --- | --- | --- | --- | --- | --- | --- | --- | --- | --- | --- | --- | --- | --- | --- | --- | --- | --- |
|  |  |  | **Routine maintenance** | | | | | **Social-oriented reflection** | | | | **Prosocial involvement** | | | | | **Social bonding** | | |
|  |  |  | **Age 1** | **Age 2** | **Age 3** | | | **Age 1** | **Age 2** | | **Age 3** | **Age 1** | **Age 2** | | **Age 3** | | **Age 1** | **Age 2** | **Age 3** |
|  |  |  | **β** | **β** | **β** | | | **β** | **β** | | **β** | **β** | **β** | | **β** | | **β** | **β** | **β** |
| Sex | | | .08 | .10* | .09 | | | .09* | .01 | | -.05 | .11 | .00 | | .07 | | .06 | .12* | .09 |
| Age | | | -.07 | .04 | -.02 | | | .12* | .03 | | -.09 | -01 | .07 | | -.01 | | .07 | .10* | -.11* |
| SES | | | -.03 | -.05 | .05 | | | .00 | .02 | | .02 | -.07 | .06* | | .25* | | -.01 | .02 | .16* |
| ***Main effects*** | | | | | | | | | | | | | | | | |  |  |  |
| **COVID-19 stressors** | | | | | | | | | | | | | | | | |  |  |  |
| Close contagion | | | .00 | -.03 | .13 | | | -.05 | -.02 | | -.06 | .03 | .00 | | .00 | | -.05 | -.06 | .08 |
| Close death | | | -.05 | .08 | .09 | | | .01 | .01 | | .13 | .00 | -.01 | | .18 | | -.03 | .00 | -.29*** |
| Economic impact | | | -.10* | -.09 | -.04 | | | -.05 | .10 | | -.18* | -.12* | .05 | | -.02 | | -.04 | .04 | -.03 |
| Fear of the future | | | .03 | .06 | .06 | | | .12* | .03 | | .13 | .07 | .07 | | .15* | | .08 | .03 | .08 |
| **Parent resilience** | | | | | |  | | | | | | | | | | | |  |  |
| Trait resilience | | | .25*** | .13* | .17* | | | .11* | .04 | | .05 | .21*** | .16* | | -.13* | | .15* | .07 | .01 |
| **Child coping** | | | | | | | | | | | | | | | | | |  |  |
| Engagement | | | .26*** | .32*** | .22* | | | .29*** | .35*** | | .30*** | .22*** | .23*** | | .29*** | | .17* | .13* | .11 |
| Disengagement | | | -.15* | -.11* | -.10 | | | -.07 | .04 | | .09 | .01 | .01 | | .15* | | .01 | .06 | .07 |
| **R^2^** | | | .18*** | .16*** | .12 | | | .15*** | .13*** | | .18*** | .07* | .11* | | .17*** | | .07* | .05* | .16*** |
| ***Interactive effects* (I)** | | | | | | | | | | | | | | | | |  |  |  |
| **Contextual interaction** | | | | | | | | | | | | | | | | |  |  |  |
| Engag x Contagion | | | .00 | -.13* | .00 | | | .02 | -18* | | .03 | .00 | -.22*** | | -04 | | .06 | -.04 | -.03 |
| Engag x Death | | | -.04 | .10 | -.05 | | | -.06 | .17* | | -.01 | -.02 | .15* | | -.01 | | -.04 | .05 | -.04 |
| Engag x Econ. impact | | | .13 | -.05 | -08 | | | .01 | -.05 | | -.14 | .10 | -.09 | | -.14 | | .18* | -.12 | .05 |
| Engag x Fear of the future | | | .27* | .27* | -14 | | | .12 | .23 | | -.01 | .15 | .31* | | .05 | | .05 | .05 | -.08 |
| **R^2^** | | | .11* | .14* | .20 | | | .09* | .17* | | .27* | .07* | .20* | | .23* | | .06 | .09 | .19* |
| ***Interactive effects* (II)** | | | | | | | | | | | | | | |  | |  |  |  |
| **Coping interaction** | | | | | | | | | | | | | | |  | |  |  |  |
| Engag x Res | | | -.07* | .00 | -.05 | | | -.05 | -.13* | | -.09 | -.01 | -.10 | | -.06 | | -.03 | -.03 | .14 |
| **R^2^** | | | .15*** | .14*** | .11* | | | 14*** | .16*** | | .22*** | .12*** | .12* | | .20*** | | .07* | .05* | .10* |
| ***Note****.* *p<.05, **p<.01, ***p<.001.  Age 1= group of 3-6-year-olds, *N= 481*; Age 2=group 2 of 7-9- year-olds, *N= 393*; Age 3: group of 10-12- year-olds*, N=248*. | | | | | | | | | | | | | | | | | |  |  |
|  |  |  | |  | | | | | |  | | | |  | |  |  |  |  |
